# Academic orientation and alcohol-related harm among adolescents: Does the inclusion of a disadvantaged group add to the social gradient?

**DOI:** 10.1186/s12889-024-20485-x

**Published:** 2024-10-28

**Authors:** Siri Thor, Jonas Landberg, Patrik Karlsson, Isabella Gripe

**Affiliations:** 1 Department of Clinical Neuroscience, Karolinska Institutet, Stockholm, Sweden; 2 Swedish Council for Information on Alcohol and Other Drugs, CAN, Östgötagatan 90, Stockholm, 116 64 Sweden; 3 Department of Social Work, Stockholm University, Stockholm, Sweden; 4 Department of Public Health Sciences, Stockholm University, Stockholm, Sweden

**Keywords:** Alcohol use, Adolescents, Alcohol-related harm, Social gradient, Students, Socio-economic status

## Abstract

**Background:**

This study aimed to examine the social gradient in self-reported alcohol-related harm (ARH) among young alcohol consumers by including a largely overlooked group of adolescents. We also explored the extent to which such a gradient could be attributed to differential exposure or differential vulnerability to risk factors.

**Method:**

Cross-sectional survey of upper-secondary students (*n* = 2996) in Sweden. Negative binomial regressions estimated the relationship between academic orientation (higher education preparatory; HEP, vocational; VP and introductory; IP) and ARH. To assess the contribution of explanatory factors, we estimated models that included risk factors such as heavy episodic drinking (HED).

**Results:**

A graded association was observed between academic orientation and ARH, with more ARH among students in IP (IRR = 1.79) and VP (IRR = 1.43) than in HEP. Adjustments for risk factors attenuated the estimates by approximately half, but there was still 14% more ARH in VP and 50% more in IP than in HEP. The additive interaction test indicated a positive and significant interaction for students in VP who engaged in HED, versus students in HEP, who did not.

**Conclusion:**

The findings suggest a negative gradient in the association between academic orientation and ARH, where the students in IP experienced the highest levels of ARH. While differential exposure and vulnerability to HED account for a significant proportion of the excess risk among VP students, HED seems to be less important relative to other risk factors among IP students. More research is needed to identify the mechanisms underlying the elevated levels of ARH among the most disadvantaged group—students enrolled in IP.

**Supplementary Information:**

The online version contains supplementary material available at 10.1186/s12889-024-20485-x.

## Introduction

Groups with low socioeconomic status (SES) face an increased risk of alcohol-related harm (ARH), surpassing the social gradient seen in other health outcomes [1, 2]. This is partly attributed to a greater prevalence of harmful drinking in lower SES groups [3]. Nonetheless, evidence suggests that SES has a direct association with ARH, independent of drinking patterns [4].

Findings regarding a possible social gradient in ARH among youth are more inconclusive, with studies pointing in different directions in regard to the association between indicators of socio-economic status and ARH [5–7]. However, studies on the social gradient in ARH among adolescents using academic orientation as an indicator of future SES have shown that students in vocational programmes (VP) are more likely than students in higher education preparatory programmes (HEP) to experience ARH [8] use restricted substances [9, 10] and use cannabis more frequently [11].

However, these studies do not take into account that there are a growing number of adolescents not eligible for the national programs (i.e. HEP and VP) in upper secondary school. In 2019, approximately 16% of those finishing compulsory school at approximately 16 years of age were not qualified to enter upper secondary school [12]. Although most of these adolescents continue their education, they are referred to a so-called introductory programme (IP). Education in IP do not lead to a degree and there is a heavy focus on the individual study plan, due to the organization of the IP, this group is rarely included in the sampling frame in school surveys. Failing to finish upper secondary school is a common issue not limited to the Swedish setting, on the contrary, in both the US and Europe substantial proportions of adolescents drop out or leave school early [13, 14]. Non-completion of school is an important public health issue since education plays a significant role in shaping living conditions, well-being and health [15]. In fact, adolescents who fail to complete compulsory school have poorer prospects in life, including a greater risk of youth unemployment and social exclusion [16].

A study that followed students in the IP showed that, five years later, 26% were neither studying nor employed [17], factors that in turn increase the risk of alcohol use disorders [18, 19]. Considering health inequalities, it is important to assess whether this group already experiences more harm from drinking at younger ages.

The students in IP differ from those in HEP and VP not only by not having been able to complete compulsory school. Previous studies also show they have lower motivation for school, early onset of substance use, they report higher levels of truancy, lower levels of school enjoyment [20] and they have a higher risk of frequent cannabis use [21], compared with students in VP and HEP. Moreover, in the IP programme there is a higher prevalence of foreign background and low educated parents [20, 21].

Studies focusing on explaining the social gradient in ARH in the general population often focus on two key mechanisms [4, 22]. The first, differential exposure, refers to that lower SES groups tend to be disproportionally exposed to additional behavioral and social risk factors that increases the likelihood for ARH [23–25]. In other words, this suggests that the association between SES and ARH is mediated by these additional risk factors. In line with this, a study on academic orientation revealed that much of the difference in self-reported ARH could be attributed to unequal exposure to heavy episodic drinking (HED) [8]. The second mechanism, differential vulnerability, posits that the increased levels of additional behavioral and social risk factors among low SES groups may interact with alcohol use, elevating the risk for ARH at any given level or pattern of drinking [24]. This latter mechanism thus refers to effect modification of SES on the association between alcohol use and ARH, and has been the focus of less research [22].

Regarding differential exposure, students in IP seem to have an elevated exposure to several risk factors for harmful drinking and related problems. Both problematic alcohol use among parents and early-onset substance use are risk factors for high alcohol consumption [26–28] and ARH [29–33]. Furthermore, previous studies show that both truancy and HED are associated with ARH [34, 35]. Finally, in line with differential vulnerability, these additional risk factors among students in IP might interact with alcohol use, increasing the risk of ARH at the same pattern of drinking.

Measuring adolescent SES can be complicated given that adolescents are on a path toward establishing their own SES. Leaning on previous studies [8–11], we will use academic orientation as an indicator of future SES.

The overall objective of this study is to increase our understanding of the social gradient in ARH among adolescents by including IP students, a largely overlooked group of adolescents with several disadvantages, in the sample frame. To this end, we will examine whether and to what degree alcohol consumers in the IP and VP experience higher levels of ARH than their counterparts in HEP. If a relationship is found, we will assess the extent to which it may be explained by (i) differential exposure, i.e., a higher prevalence of risk factors among students in the IP and VP, such as HED, problematic alcohol consumption among parents, age at onset of substance use, etc., or (ii) differential vulnerability to HED, such that HED has a stronger association with ARH among students in IP or VP than among those in HEP.

## Data and methods

The study is based on two cross-sectional datasets. Both surveys were conducted in 2021 by the Swedish Council for Information on Alcohol and Other Drugs (CAN). Both web-based, anonymous surveys were completed in the classroom. For the questionnaire, please see supplementary material. The first dataset (1) consists of data from the 2021 iteration of the Swedish National School Survey among second-year students enrolled in national programmes (VP and HEP) in upper secondary school (17- to 18-year-olds). A stratified sampling procedure ensured that all regions in Sweden were represented, and the school class (one per school) was used as the unit when selecting the sample.

The second dataset (2) consists of data from the IP in upper secondary school. To ensure anonymity, only schools with at least 20 students enrolled in the IP were included in the sampling frame. The survey, which was offered to all students in the randomly selected IP, was designed to generate results comparable to those from the annual Swedish national school survey. Students enrolled in language introduction who recently arrived in Sweden were not included since they were unable to answer the survey in Swedish or English.

The participation rate in dataset (1) was 74% on the school/class level and the response rate on the individual level (students who were present and chose to participate) was 81% [20]. The participation rate in dataset (2) was 56% at the class level and 67% at the individual level [20]. We use listwise deletion and the analytical sample consisted of alcohol consumers (all students who stated that they had consumed at least one alcoholic beverage in the last 12 months) who had completed all of the survey items: 2096 students in HEP, 668 students in VP and 232 students in IP (see Table 1).

## Measurements

### Exposure

Information on *academic orientation* was provided by each school prior to the survey. The school classes included in the analysis were coded as either VP or HEP [in dataset (1)] or IP [in dataset (2)].

**Table 1:** Sample description - distribution of key variables across academic orientation. Among consumers

		HEP	VP	IP
Participants	(n)	2096	668	232
Alcohol-related harm	mean	2.41	3.44	4.29
*Gender*				
Men	%	42	55	62
Women	%	58	45	38
*Heavy episodic drinking*				
6 times/year or less	%	43	41	42
Once a month or more	%	25	33	31
*Recurrent truancy*				
Once a month or more often	%	10	13	29
*Family satisfaction*				
Dissatisfied	%	13	13	19
*CAST-6*				
Yes (≥ 3 problems)	%	14	21	16
*Early onset substance use*				
Onset < 14 years old	%	15	26	40
*Place of birth*				
Born in Sweden w at least 1 parent born in Sweden	%	88	89	57
*Parental SES*				
At least 1 parent studied at college	%	80	51	46

### Outcome

*Self-reported alcohol-related problems* (hereafter referred to as alcohol-related harm, ARH) were measured using the following question:

Has any of the following things happened to you in relation to your alcohol consumption during the past 12 months?

**Table:** 

1. Quarrel	2. Deliberately harmed yourself
3. Physical fight	4. Victim of robbery or theft
5. Accident or injury	6. Trouble with the police
7. Engaged in sex you regretted the next day	8. Needed go to hospital or emergency room
9. Deliberately harmed someone else	10. Driving a moped or other motor vehicle
11. Victim of violence	12. Lost money or valuables
13. Riding a moped or other motor vehicle with a drunk driver	14. Been photographed/filmed in an embarrassing or humiliating situation
15. Ruined clothes or other belongings	16. Gone swimming in deep waters
17. Problems with relations to parents	18. Problems with relations to friends

The response options ‘never’, ‘once’ and ‘twice’ were coded as 0, 1 and 2, respectively. Respondents who selected the highest value on more than 16 of the problem-related questions were deemed unreliable and excluded from the study. Respondents who did not answer a specific item was coded as 0, never, on that item. The total variable score therefore ranged from 0 to 33, with higher scores indicating more ARH. The internal reliability was good: Cronbach’s alpha = 0.86 (0.82 for HEP, 0.89 for VP, 0.92 for IP).

### Explanatory variables

*Heavy episodic drinking* (HED) was measured using a question regarding how often the student (during the past 12 months), on one continuous occasion, had consumed an amount of alcohol equivalent to at least a whole bottle of wine or four cans of beer/mixed drinks or six cans of medium-strength beer (3.5% per volume) or 18 cl of spirits. The measure intends to describe a large amount of alcohol consumed on one occasion, making it less important for respondents to know the exact amount they consumed. Possible responses were; Never, once during the last 12 month, 2–6 times during the last 12 month, Once a month, 2–3 times a month, Once or a few times a week. The responses were coded into three categories for the descriptive tables: no HED during the last 12 months, HED 6 times a year or less, and HED once a month or more often. Respondents who didn’t answer the question were excluded from the analysis and comprised *n* = 38 in the sample of alcohol consumers.

*Truancy* was measured using the question “Do you tend to skip school?”. Responses ranging between 1 (“No”) to 6 (“Yes, several times a week”). Responses ranging from once a month to several times a week were coded as recurrent truancy. Respondents who didn’t answer the question were excluded from the analysis and comprised *n* = 22 in the sample of alcohol consumers.

*CAST-6.* To investigate the extent to which students felt that their parents’ alcohol use was problematic, the Children of Alcoholics Screening Test (CAST-6) was used. This instrument consists of six questions and is a shortened version of the original 30-question test. CAST-6 identifies children who perceive their parents’ alcohol consumption as problematic equally well as does the original 30-question test [36]. CAST-6 poses six questions regarding the negative consequences of parents’ drinking. If three of these six negative consequences were met, the student was coded as having at least one parent with problematic alcohol use, which is an established cutoff [36].

*Early-onset substance use* was defined as the use of any of the substances stated below before the age of 14 years and was measured using five questions: How old were you when (if ever) you did the following things for the first time? (1) Drank at least one glass of alcohol, (2) Got drunk on alcohol, (3) Smoked a cigarette, (4) Used moist snuff, (5) Used marijuana or hashish. Individuals who responded yes to any of these questions were defined as having an early onset of substance use. Respondents who didn’t answer any of the questions were excluded from the analysis and comprised *n* = 71 in the sample of alcohol consumers.

*Family satisfaction* was measured using the question ‘How satisfied are you with your relationship with your family in general?’ Responses ranging between (1) Very satisfied to (5) Very dissatisfied. Those who reported being dissatisfied, very dissatisfied or neither satisfied nor dissatisfied were coded as being dissatisfied with their relationship with their family. Respondents who didn’t answer the question were excluded from the analysis and comprised *n* = 26 in the sample of alcohol consumers.

*Parental SES* was measured using parents’ educational attainment, as reported by the student. It was created using two questions: Has your father/mother studied at university or college? The questions were combined and coded into two categories: at least one parent has studied at college and neither parent has studied at college. Excluded from the analysis were students who did not respond or did not know for either of the parents. These responses comprised *n* = 9 in the sample of alcohol consumers.

Place of birth was measured using three questions “I’m born in…/Mom is born in…/Dad is born in…” with the response options (1) In Sweden, (2) In another Nordic country, (3) In another European country, (4) In a country outside Europe, (5) I don’t know. Individuals responding they were born in Sweden and either mom or dad were born in Sweden were coded as being born in Sweden with at least one parent born in Sweden. Students who didn’t know or didn’t answer were excluded and comprised *n* = 64 in the sample of alcohol consumers.

*In addition*,* all analyses were adjusted for gender*, measured using a question where the students could choose between male, female and other gender identities. The respondents who chose one of the two first alternatives were included in the study. Respondents who didn’t answer or chose “other identity” were excluded and comprised *n* = 28 in the sample of alcohol consumers.

### Statistical analyses

Since the outcome was a count variable with an over dispersed distribution (mean = 2.80, variance = 19.44), negative binomial regressions were used to assess the association between academic orientation and ARH. The results section presents incidence rate ratios (IRRs) and 95% confidence intervals.

To explore the social gradient in ARH, we estimated the first model, which included academic orientation and gender (included in all the models). To assess the contribution of the explanatory factors, we estimated models with separate adjustments for HED (Model 2), truancy (Model 3), early-onset substance use (Model 4), family dissatisfaction and CAST-6 score and (Model 5) place of birth and parental SES (Model 6). The final model included all the explanatory variables.

The effect of the explanatory variables on the association between academic orientation and ARH was estimated by calculating the attenuation (in percentages) between the estimate in the crude model with each of the consecutive models, the following formula was used: (*IRRcrude-IRRadjusted)/(IRRcrude-1) × 100*.

Since the students in dataset (1) were clustered in school classes and the students in dataset (2) were clustered in schools, we needed to use a procedure that relaxes the usual assumption of independence between observations in order to not underestimate the standard errors [37]. We chose the vce (cluster) option in Stata version 17.

To test for effect modification, we tested for interactions between academic orientation and HED, both on the multiplicative and the additive scales.

Multiplicative interactions were tested by including an interaction term between academic orientation and HED (academic orientation × HED) and performing a Wald test. To test for additive interactions, we included a variable that combined information on academic orientation and HED. We then calculated the relative excess risk due to interaction (RERI) using the formula *RERI = IRR*_*++*_*– IRR*_*+−*_*– IRR*_*−+*_*+ 1.* This measure assesses whether the IRRs of the combined exposures (i.e., IP students and HED, *IRR*_*++*_) are greater or smaller than the sum of the IRRs for each exposure individually (i.e., IP students with no HED, *IRR*_*+−*_ and not IP student and HED, *IRR*_*−+*_).

We also calculated the proportion of ARH among those with both exposures that was attributable to their interaction; AP (attributable proportion) = *RERI/IRR*_*++*_.

## Results

### Descriptive statistics

Table 1 presents the distributions of key variables across academic orientations. The mean number of ARH increased across academic orientations; students in HEP reported an average of 2.41 problems, those in VP reported 3.44, and those in IP reported 4.29 problems. A similar gradual increase was shown for truancy and early-onset substance use. However, engaging in HED at least once a month during the last 12 months was equally common in VP and IP and less common in HEP. The proportion of students who were not satisfied with their family relationship was highest in IP; it was lower and similar in the other two programmes. The proportion of students reporting problematic alcohol use among parents (CAST-6) was highest in VP. Furthermore, the lowest proportion of students reporting being born in Sweden with at least one parent born in Sweden was found in IP, likewise the lowest proportion of having at least one parent who studied at college was also found in IP.

### Regression analysis

The results of the negative binomial regression are shown in Table 2. The crude model (only adjusted for gender) revealed a negative graded association between academic orientation and ARH, where the amount of ARH increased with each step down in academic orientation. IRRs of 1.43 (95% CI 1.21 to 1.69) and 1.79 (CI 95% 1.41 to 2.26) implies that the students in VP experience 43% higher levels of harm, and students in IP experience 79% higher levels of harm, compared to students in HEP.

**Table 2:** Association between academic orientation and alcohol-related harm, among consumers. Negative binomial regression

		IRR^a^	95% CI		Attenuation^b^
	Crude model (adjusted for gender)				
	Vocational	1.43	1.21	1.69	
	Introductory	1.79	1.41	2.26	
+	HED (model 2)				
	Vocational	1.23	1.07	1.41	47
	Introductory	2.00	1.52	2.63	(-)27
+	Truancy (model 3)				
	Vocational	1.35	1.14	1.61	19
	Introductory	1.44	1.11	1.88	44
+	Early onset substance use (model 4)				
	Vocational	1.27	1.07	1.50	37
	Introductory	1.38	1.08	1.77	52
+	CAST6 + family satisfaction (model 5)				
	Vocational	1.35	1.14	1.60	19
	Introductory	1.66	1.30	2.12	16
+	Place of birth + Parental SES (model 6)				
	Vocational	1.47	1.24	1.75	(-)9
	Introductory	1.84	1.46	2.33	(-)6
+	Combined/full model				
	Vocational	1.14	0.99	1.32	67
	Introductory	1.50	1.10	2.03	37

HED, heavy episodic drinking, CAST6, The children of alcoholics screening test, 6 items. CI, Confidence interval

a) The IRR (incidence rate ratio) for alcohol-related harm when adding variables separately to the crude model

b) Percentage attenuation (IRRcrude-IRRadjusted)/(IRRcrude-1) × 100

Adjusting for HED (Model 1) attenuated the difference in ARH between students in VP versus HEP by 47% (to IRR 1.23, 95% CI 1.07 to 1.41), but no attenuation was revealed for students in IP (IRR 2.00, 95% CI 1.52 to 2.63).

Adjusting for truancy in Model 3 and family satisfaction and CAST-6 in Model 5 attenuated the estimates for students in both VP and IP compared to HEP between 16 and 44%. Adjusting for place of birth and parental SES (Model 6) did not have an attenuating effect on the association on either VP or IP.

Adjusting for early-onset substance use had the largest attenuating effect, with an estimated 37% reduction in VP and 52% in IP.

The full model indicated 14% higher levels of ARH among VP students (IRR 1.14, 95% CI 0.99 to 1.32) and 50% higher among IP students (IRR 1.50, 95% CI 1.10 to 2.03) compared to students in HEP. Consequently, adjusting for all explanatory variables resulted in a 67% reduction of ARH in the VP and a 37% reduction in the IP, compared to the crude model.

### Interaction analysis

Table 3 presents the IRRs for ARH for a measure that combines academic orientation and HED. Students in VP and IP who did not engage in HED experienced more ARH than did their counterparts in HEP. This was particularly evident among IP students without HED, who reported almost three times more problems than HEP students without HED (IRR = 2.75). Furthermore, a gradual increase in the association was also visible among students who engaged in HED across the programmes. Students in HEP who engaged in HED reported five times more ARH than did those who did not, and the estimates for students in VP and IP who engaged in HED were even greater, with the latter group reporting eight times more ARH than non-HED students in HEP (Table 3). The additive interaction between programme and HED was estimated by calculating the RERI for VP and IP versus HEP. According to the unadjusted model, the RERI estimates indicated a positive additive interaction for both VP and IP and HED compared to HEP with no HED. The estimates suggested that 1.63% of the IRRs for students in VP and 1.32% of the IRRs for students in IP who engaged in HED were attributed to an interaction. This implies that 24% (AP = 0.24) in VP and 16% (AP = 0.16) in IP was attributable to being exposed to both HED and a lower academic orientation. However, the RERI and AP estimates were statistically significant only for the double-exposure VP and HED (1.63 95% CI 0.56 to 2.69). According to the full model, neither the RERI nor the AP was significant for either combination. We also investigated whether HED and academic orientation interacted on the multiplicative scale and found no statistically significant interaction (Wald test, *p* value 0.0579).

**Table 3:** Association between the combined variable of academic orientation and HED, and alcohol-related harm. Negative binomial regression, RERI and AP

	IRR^a)^	95% CI	
Crude model^a)^			
Higher education preparatory/no HED	ref.	ref.	ref.
Vocational/No HED	1.18	0.75	1.85
Introductory/No HED	2.75	1.53	4.94
Higher education preparatory/HED	5.00	3.84	6.51
Vocational/HED	6.81	5.10	9.09
Introductory/HED	8.07	5.77	11.29
Double exposure; introductory + HED			
RERI	1.32	(-)0.87	3.50
AP	0.16	(-)0.08	0.41
Double exposure; vocational + HED			
RERI	1.63	0.56	2.69
AP	0.24	0.11	0.37
Full model^b)^			
Higher education preparatory/no HED	ref.	ref.	ref.
Vocational/No HED	1.27	0.74	1.85
Introductory/No HED	2.30	1.32	4.01
Higher education preparatory/HED	4.39	3.37	5.71
Vocational/HED	5.28	3.90	7.14
Introductory/HED	4.94	3.42	7.15
Double exposure; introductory + HED			
RERI	(-)0.74	(-)2.40	0.91
AP	(-)0.15	(-)0.50	0.20
Double exposure; vocational + HED			
RERI	0.73	(-)0.21	1.66
AP	0.14	(-)0.03	0.30

HED, Heavy episodic drinking. RERI, The relative excess risk due to interaction. AP, Attributable proportion. The IRR (incidence rate ratio) for alcohol-related harm. CI, Confidence interval

a) Adjusted for gender

b) Adjusted for gender, truancy, early onset substance use, family satisfaction, Children of Alcoholics Screening Test (CAST6), Parental SES and place of birth

## Discussion

This study set out to increase our understanding of the social gradient in ARH among adolescents by including a group of adolescents, with several disadvantaged, previously overlooked in alcohol research. Our results revealed a negative association between academic orientation and ARH, where the highest levels of harm were found among students enrolled in IP, followed by students in VP. Our study thus adds to previous literature by showing that the most disadvantaged group of adolescents also experiences the highest levels of ARH [2].

Next, we assessed to what degree the social gradient in ARH could be attributed to unequal exposure to HED and other risk factors. Together, these explanatory factors account for approximately half of the differences in ARH across academic orientations. Unexpectedly, adjustment for HED did not attenuate the levels of ARH among students in IP relative to those in HEP. However, among students in the VP, the estimate was attenuated by 47%, which is in line with results in adults [22]. Still, part of the negative gradient remained unexplained, as ARH was 14% higher among VP students and 50% higher among IP students, than students in HEP. Adjustment for truancy and early-onset substance use resulted in the largest attenuations in both programmes. This observation aligns with prior research, finding associations between both truancy [34] and early substance use [38, 39] and problems in relation to alcohol consumption.

When examining the mechanism of differential vulnerability, we found some indications that the association between HED and ARH was stronger among students in IP and VP. The results revealed a potential excess risk due to interaction for students in VP and IP who engaged in HED, in comparison to students in HEP, which agrees with the findings of other studies [23]. However, the interaction was significant for only the unadjusted analysis and for VP students, for which 24% of the IRR for ARH were attributable to the interaction effect of being exposed to both the programme and engaging in HED.

The lack of statistical significance for additive interaction among IP students could be due to low statistical power [40]. However, the RERI indicated that only 16% of the excess risk for this group is due to the interaction of being exposed to both the programme and engaging in HED. This low AP may be partly explained by the fact that IP students who did not engage in HED also demonstrated a large excess risk, with ARH levels almost three times higher than those in the HEP group.

Taken together, our results suggest that the elevated levels of ARH among VP students can be partly attributed to differences in drinking patterns, as well as differential vulnerability to HED, which is in line with previous findings among adults [22]. However, even though there were some indications of excess risk due to interaction for students in IP who engaged in HED, neither differential exposure nor differential vulnerability to HED could explain the higher levels of ARH among students in IP. This suggests that these students, to a greater extent, experience the harm included in our outcome, irrespective of their drinking. In fact, almost half of the elevated levels of ARH among IP students could be attributed to differential exposure to risk factors other than HED. It is plausible that this group more frequently finds themselves in “problematic” contexts, such as altercations with peers or police or being the victim of violence. Consequently, their elevated levels of ARH could be due to an increased risk of experiencing such problems in the first place, i.e. also without the involvement of alcohol. This suggests that, even with similar levels of drinking or HED, this group may report more problems than their less disadvantaged peers, due to greater exposure to other risk factors. Given that previous studies including this group, to our knowledge, are very few, this is a matter that needs further attention.

Finally, the Swedish school system provides an interesting setting where low-performing students in upper secondary school are gathered, still present within the school setting and thereby possible to reach through the school. The school arena may represent a last chance to effectively reach adolescents with broader interventions. Considering the results of the study suggesting that disadvantaged students in particular have a heightened risk of ARH, it is important that future research focus on identifying the specific pathways and mechanisms involved in this relationship. With a fuller understanding of these factors, interventions can be developed targeting adolescents with disadvantages such as school failure.

Strengths and limitations.

The Swedish national school surveys have been proven to have both a high response rate and good sociodemographic coverage [41]. However, the nonresponse rate was greater in our additional survey of the IP. A study of Swedish national school surveys showed that students who were not present at the time of the survey consumed more alcohol, drugs and tobacco than did their classmates [42]. This is likely also true for the IP but should not affect the overall results of this study, it might however imply that we have underestimated the association between programme and ARH. Furthermore, in all studies relying on self-reported answers, there is always a risk related to under/over-estimating one’s answers or to the respondent not remembering correctly [43]. To minimize this type of bias, the survey was conducted with a school personnel present and with a strong emphasis on each students anonymity [44]. Information on academic orientation was collected through the school and was therefore not a subject of that type of bias. The use of a cross-sectional design hinders the ability to determine the causal relationship between academic orientation and ARH. We cannot rule out that problems related to alcohol existed before upper secondary school with possible effects on the individual’s academic trajectory. To address this issue, future research should employ a longitudinal design to more definitively answer this question.

## Conclusions

Our findings revealed a negative gradient in the association between academic orientation and ARH, where the students in IP experienced the highest levels of ARH. While differential exposure and vulnerability to HED account for some of the excess risk among VP students, HED seems to be less important than other risk factors among IP students. More research is needed to disentangle the mechanisms underlying the elevated levels of ARH among the most disadvantaged group—students enrolled in IP.

## Electronic supplementary material

Below is the link to the electronic supplementary material.

Supplementary Material 1

## Data Availability

The datasets generated and analysed during the current study are not publicly available but are available from the corresponding author on reasonable request.
