# Supplementary material for: Academic orientation and alcohol-related harm among adolescents: Does the inclusion of a disadvantaged group add to the social gradient?
Source: BMC Public Health. 2024 Oct 28;24:2981. doi: 10.1186/s12889-024-20485-x (PMC11520370; doi:10.1186/s12889-024-20485-x)
Supplement: Supplementary file 1 — Supplementary Material 1 [file 12889_2024_20485_MOESM1_ESM.pdf]

C.A.N

Hi!

This survey is performed to increase knowledge regarding young people's experiences of alcohol, tobacco, and narcotics, among other things. By responding to the questions, you will help us find out more about the current situation for young people.

The survey is completely anonymous and we guarantee that your responses cannot be traced back to you.

Participation is voluntary. If there is any question that you cannot or do not want to respond to, you can simply skip it.
When you have filled out your responses, click on "Submit" in the final tab (25). If you want to send in the questionnaire before completion or do not want to participate at all, you do this in the same way.

Thank you for participating!

Are you…?

- ◯ Male
- ◯ Female
- ◯ Other gender identity

In what year were you born?

- ◯ 2002 or earlier
- ◯ 2003
- ◯ 2004
- ◯ 2005
- ◯ 2006 or later

Have your parents/guardians studied at a university or higher education institution?

Tick one box for each sub-question.

a) Father/other guardian	◯ Yes	◯ No	◯ I don't know
b) Mother/other guardian	◯ Yes	◯ No	◯ I don't know

How satisfied are you usually with…

	Very satisfied	Satisfied	Neither satisfied nor dissatisfied	Dissatisfied	Very dissatisfied
a) …your family's financial situation?	◯	◯	◯	◯	◯
b) …your health?	◯	◯	◯	◯	◯
c) …yourself?	◯	◯	◯	◯	◯

Have you ever smoked a cigarette?

Tick one or more boxes.

- ☐ No
- ☐ Yes, in the last 30 days
- ☐ Yes, in the last 12 months
- ☐ Yes, more than 12 months ago

Do you still smoke?

- ○ No, I've just tried it
- ○ No, I have quit
- ○ Yes, every day
- ○ Yes, almost every day
- ○ Yes, but only at parties
- ○ Yes, but only occasionally

Have you ever used snuff?

Tick one or more boxes.

- ☐ No
- ☐ Yes, in the last 30 days
- ☐ Yes, in the last 12 months
- ☐ Yes, more than 12 months ago

Do you still use snuff?

- ○ No, I've just tried it
- ○ No, I have quit
- ○ Yes, every day
- ○ Yes, almost every day
- ○ Yes, but only at parties
- ○ Yes, but only occasionally

Have you ever drunk alcohol?

Do not include t ex soft cider, low-alcohol beer, or other beverages with an alcohol content by volume of less than 2.26%.

Tick one or mor boxes.

- ☐ No
- ☐ Yes, in the last 30 days
- ☐ Yes, in the last 12 months
- ☐ Yes, more than 12 months ago

How often do you drink alcohol?

- ○ Not within the last 12 months
- ○ Once a month or less often
- ○ 2–4 times a month
- ○ 2–3 times a week
- ○ 4 times a week or more often

About how many "units" do you drink when you drink alcohol

See examples of "units" below.

- ○ 1–2
- ○ 3–4
- ○ 5–6
- ○ 7–9
- ○ 10 or more

How often do you drink six such "units" or more at a single occasion?

- ○ Never
- ○ Less often than once a month
- ○ Every month
- ○ Every week
- ○ Daily or almost daily

Think back over the last 12 months.

How often have you, at a single occasion, drunk an amount of alcohol corresponding to at least four large cans of strong beer/hard cider or 25 cl of hard liquor or six large cans of medium-strength beer?

- Once a week or more often
- 2–3 times a month
- Once a month
- 2–6 times in the last 12 months
- Once in the last 12 months
- Not within the last 12 months

Has any of the following happened to you when you have been drinking alcohol during the last 12 months?

Tick one box for each sub-question.

	No	Once	Twice or more
a) Gotten into an argument			
b) Gotten into a fight			
c) Had an accident or been injured			
d) Intentionally harmed yourself			
e) Intentionally harmed someone else			
f) Been exposed to violence			

	No	Once	Twice or more
g) Lost money or valuables			
h) Ruined possessions or clothes			
i) Experienced problems in your relationship with your parents			
j) Experienced problems in your relationship with your friends			
k) Had sex that you regretted the next day			
l) Been a victim of theft or robbery			

	No	Once	Twice or more
m) Had an altercation with the police			
n) Had to visit a hospital or emergency room			
o) Driven a moped, car or other motor vehicle			
p) Ridden a moped, car or other motor vehicle with a driver who was drunk			
q) Gone swimming in deep water			
r) Been photographed or filmed in an embarrassing or offensive situation			

Have you ever used anabolic-androgenic steroids (AAS) without a doctor's prescription?

Tick one or more boxes.

- ☐ No
- ☐ Yes, in the last 30 days
- ☐ Yes, in the last 12 months
- ☐ Yes, more than 12 months ago

Have you used prescription sleeping pills or sedatives without a doctor's prescription?

Examples include Stesolid/Valium, Imovane, Xanor/Xanax, and benzodiazepines.

Tick one or more boxes

- ☐ No
- ☐ Yes, in the last 30 days
- ☐ Yes, in the last 12 months
- ☐ Yes, more than 12 months ago

Have you used prescription painkillers without a doctor's prescription

Examples include tramadol, Citodon/co-codamol, oxycodone, and morphine.

Tick one or more boxes.

- ☐ No
- ☐ Yes, in the last 30 days
- ☐ Yes, in the last 12 months
- ☐ Yes, more than 12 months ago

Have you used prescription stimulants without a doctor's prescription?

Examples include ADHD medication like Ritalin, Concerta, Attentin, and Elvanse.

Tick one or more boxes.

- ☐ No
- ☐ Yes, in the last 30 days
- ☐ Yes, in the last 12 months
- ☐ Yes, more than 12 months ago

Have you, in the last 12 months, ever been offered to try or buy narcotics?

Narcotics include, for example, hashish, marijuana, amphetamine, cocaine, and heroin.

- ○ Yes
- ○ No

Have you ever used narcotics?

Narcotics include, for example, hashish, marijuana, amphetamine, cocaine, and heroin.

Tick one or more boxes.

- ☐ No
- ☐ Yes, in the last 30 days
- ☐ Yes, in the last 12 months
- ☐ Yes, more than 12 months ago

The following questions are about sniffing and narcotics

Which kind or kinds of narcotics have you used?

Tick one or more boxes.

- ☐ Hashish
- ☐ Marijuana
- ☐ Spice or similar blends for smoking
- ☐ Amphetamine
- ☐ Cocaine
- ☐ Prescription sleeping pills or sedatives without a doctor's prescription (Examples include Stesolid/Valium, Imovane, Xanor/Xanax, benzodiazepines)
- ☐ Prescription painkillers without a doctor's prescription (Examples include tramadol, Citodon/co-codamol, oxycodone, and morphine)
- ☐ Prescription stimulants without a doctor's prescription? (Examples include ADHD medication like Ritalin, Concerta)
- ☐ Ecstasy
- ☐ LSD, magic mushrooms, or other hallucinogens
- ☐ Heroin
- ☐ Other type:
- ☐ I don't know

How many times have you used…

	Never	Once	2–4 times	5–10 times	11–20 times	21–50 times	More than 50 times
a) …hashish and/or marijuana?	○	○	○	○	○	○	○
b) …narcotics other than hashish/marijuana?	○	○	○	○	○	○	○

In the last 12 months, approximately how often have you used narcotics?

- ○ Every day
- ○ Every other day
- ○ Twice a week
- ○ Once a week
- ○ 2–3 times a month
- ○ Once a month
- ○ 2–6 times in the last 12 months
- ○ Once in the last 12 months
- ○ Not within the last 12 months

How old were you when you did the following things for the first time (if ever)?

Tick one box for each sub-question.

	Never	11 years or younger	12 years	13 years	14 years	15 years	16 years	17 years	18 years or older
a) Had at least one unit of alcohol	○	○	○	○	○	○	○	○	○
b) Became intoxicated from alcohol	○	○	○	○	○	○	○	○	○
c) Smoked a cigarette	○	○	○	○	○	○	○	○	○
d) Used snuff	○	○	○	○	○	○	○	○	○
e) Used marijuana or hashish	○	○	○	○	○	○	○	○	○

Have you ever gambled?

For example bought lottery tickets, played poker for money, placed a sports bet or similar.

Tick one or more boxes.

- ☐ No
- ☐ Yes, in the last 30 days
- ☐ Yes, in the last 12 months
- ☐ Yes, more than 12 months ago

How satisfied are you, in general, with your relationship with your family?

- ○ Very satisfied
- ○ Satisfied
- ○ Neither satisfied nor dissatisfied
- ○ Dissatisfied
- ○ Very dissatisfied

How do you feel about school?

- ○ I like it a lot
- ○ I think it's alright
- ○ I feel neutral about it
- ○ I do not like it very much
- ○ I do not like it at all

Do you ever skip class?

- No
- Yes, once or twice a term
- Yes, once a month
- Yes, 2–3 times a month
- Yes, about once a week
- Yes, several times a week

Thank you!

If there is anything you would like to add, please write it in this box:

This is the end of the form. Click on the button “Submit” to finalize the process.

Thank you for your participation!

If you want advice and support or have any questions about alcohol or other drugs, you can find information at www.drugsmart.se, www.bris.se, www.umo.se, and www.droghjalpen.se. You can read more about CAN at www.can
